# Investigating the Influence of Laser-Etched Straight and Wavy Textures on Grinding Efficiency and Tool Quality of WC–Co Carbide Cutting Tools

**DOI:** 10.3390/ma18030528

**Published:** 2025-01-24

**Authors:** Chao Li, Tielin Li, Xiaohong Zhang, Tianzhongsen He, Linzhi Su, Dongdong Wen, Sizhen Shao, Xiao Cai, Qingzheng Cao

**Affiliations:** 1 College of Mechanical Engineering, Hunan Institute of Science and Technology, Yueyang 414006, China; 18907309755@163.com (T.L.); jansbomb@126.com (X.Z.); m13907420357@163.com (T.H.); slinz953881908@163.com (L.S.); ifdong@163.com (D.W.); 15197959366@163.com (S.S.); feizhouheiba@163.com (X.C.); caoqingzheng2024@163.com (Q.C.); 2 Key Laboratory of Intelligent Manufacturing and Service Performance Optimization of Laser and Grinding in Mechanical Industry, Yueyang 414006, China

**Keywords:** tungsten–cobalt alloy, laser surface texturing, surface integrity, surface topography, edge quality

## Abstract

WC–Co cemented carbide has been widely used as machining tool material due to its good mechanical properties. Grinding is an important process in the manufacture of cemented carbide tools. When grinding tools, there are problems such as excessive grinding force, small chip space, and poor lubrication and cooling performance, which in turn contribute to surface defects such as burrs, burns, and even edge damage such as edge chipping. These problems constrain the use of carbide tools, so that the cutting force is unstable and the machining surface quality is poor when the tool is in service. In this paper, straight-line and wavy-texture patterns were designed and formed on the surface of WC–Co tools using a picosecond laser. Grinding experiments were conducted on the ablated tool using a resin-bonded diamond wheel, and surface morphology, roughness, grinding force, and cutting edge quality were evaluated. Finally, turning experiments were conducted to compare the cutting performance of the tools after conventional and laser-assisted grinding. The experimental results showed that the tools with wavy texture showed superior surface and cutting edge quality, with 53.7% and 51.2% reduction in normal and tangential grinding forces, respectively, and 66.6% maximum reduction in edge chipping for the wavy textured tools. Therefore, this study not only reveals the advantages of laser-assisted grinding in machining WC–Co cutting tools, but also provides a valuable theoretical basis for realizing high-efficiency and low-loss tool machining.

## 1. Introduction

With the rapid development of the modern manufacturing industry, the requirements for the performance of tool materials are only increasing. As a tool material with excellent performance, WC–Co cemented carbide has been widely used in the field of cutting and machining due to its high hardness, high wear resistance, and good thermal stability [1,2]. However, due to the high hardness and brittleness of WC–Co alloy materials, a series of micro-damages, such as chip surface attachment, edge chipping, sub-surface exposure, and WC crystal fragmentation, are easily produced during machining, thus limiting the application of WC–Co tools [3,4,5]. The blanking to final shaping of a tool is an important process to ensure its performance and longevity. This process not only accurately shapes the geometry and dimensions of the tool, but also directly relates to the performance of the tool in practical applications. In the cutting and machining of Carbon Fiber Reinforced Composites (CFRP) bars, the quality of the tool edge becomes a key factor in determining the machining surface quality and machining efficiency. Therefore, improving the quality of the cutting edge of the tool is essential to optimize its overall performance in turning CFRP bars. In machining WC–Co cemented carbide, super-abrasive wheel grinding has become a major method [6]. In the process of grinding cemented carbide using traditional grinding wheels, the grinding fluid cannot effectively enter the grinding zone, and a large amount of grinding chips cannot be discharged in time, which easily leads to the material at the chipped edge being processed again by the grinding wheel; the cracks extend from the WC phase to the CO phase, and a large amount of WC crystals are squeezed out to form a large notch, which results in the quality of the tool cutting edge not being able to meet the requirements [7]. Although existing research has explored advancements in grinding processes, it has frequently overlooked the urgent necessity of enhancing chip removal and cooling mechanisms during the grinding operation. Addressing these challenges is crucial for mitigating re-damage to the workpiece caused by grinding chips, enhancing cooling and lubrication, and ultimately minimizing edge chipping of the cutting tool.

Numerous scholars have conducted in-depth studies on improving the edge quality of carbide tools. Cruz et al. [8] investigated the effects of using grinding wheels with different bonding agents and different chip thicknesses on the surface quality and edge performance of carbide tools. The results showed that resin-bonded grinding wheels significantly improved the surface finish and edge quality of carbide tools compared to glass-bonded grinding wheels. In addition, the surface quality of the tool is adversely affected when the chip thickness is large. Denkena et al. [9] used a cup-type grinding wheel with smaller grit size for precision grinding of PCBN tools, and the results of the study showed that smaller grits are beneficial for improving the edge quality of the tool. Ventura et al. [10] utilized diamond-cup-type grinding wheels with grit sizes of 15 µm and 46 µm for grinding experiments to explore in depth the effect of grit size on tool edge quality. It was found that lower surface roughness and edge chipping values were obtained for grinding carbide tools with small grits, while higher hardness and comprehensive residual stresses were produced when using coarse grits. However, the above research mainly focuses on the optimization of grinding process parameters, and does not fundamentally solve the problem that grinding chips cannot be discharged in time due to the difficulty of grinding fluid entering the grinding area effectively.

In recent years, the use of texturized runners has been proposed to improve the flow properties of grinding fluids, making it easier for grinding chips to be discharged. There are various methods for processing texturized runners, such as laser ablation [11], electrochemical etching [12], micro- and nano-imprinting [13], chemical etching [14], and self-assembly technology [15]. Compared with other texturization processing methods, laser ablation has the advantages of high precision, wide applicability, and non-contact processing [16,17,18], and is regarded as one of the most promising texturization processing methods. Cheng et al. [19] designed four surface textured by comparing normal grinding and laser-assisted grinding. They found that the laser-assisted grinding of tungsten alloys resulted in improved surfaces, with a reduction in surface roughness by 0.023–0.204 µm, normal grinding forces by 49.91–59.46%, and tangential grinding forces by 44.11–58.49%. Zhang et al. [20] used CBN grinding wheels to grind the surface of Ti-6Al-4V with a laser-textured surface, comparing six textured patterns to Ti-6Al-4V without textured patterns. The study found that the laser-textured surface significantly reduced the grinding force by 45–56% and the grinding temperature by 41–52%. Azarhoushang et al. [21] found that applying two laser ablation patterns of parallel lines on a silicon nitride workpiece significantly reduced tangential and normal grinding forces, while also slightly improving surface roughness. However, the variety of patterns remains limited. In a related study, Tshabalala et al. [22] demonstrated that enhancing material removal with a pulsed laser on silicon nitride is associated with increased lateral overlaps and pulse energy. K. Johannners et al. [23] performed cutting experiments on SAE 1045 plain carbon steel by laser texturing on the front corner face of the tool. They showed based on SEM microstructural examination that the chip mobility was provided due to the concave nests formed by laser texturing. T. Sugihara et al. [24] succeeded in creating recessed textured surfaces with different sizes and arrangement patterns on the front corner face of the tool for subsequent milling experiments on carbon steel. They found that the textured surfaces could act as microfluidic channel for milling chips, thus enhancing the surface quality of carbon steel. It has been proved that the surface microfluidic channel obtained by laser ablation is effective in solving the problems arising in machining. Similarly, it is also a feasible method to prepare microfluidic channels on the surface of WC–Co cemented carbide tools to improve the grinding lubrication conditions, enhance the chip removal ability, and then reduce the grinding force and improve the edge quality.

In this study, a laser-assisted grinding process was used to grind WC–Co carbide tools with a resin-bonded diamond wheel. Firstly, the surface of the WC–Co carbide tool was ablated using an ultrafast picosecond pulsed laser to obtain two different laser texture patterns. The overall morphology of the laser texture patterns after laser ablation was observed and analyzed. Then, the grinding force, surface morphology, surface roughness, and edge quality of WC–Co carbide tools with different laser texture patterns under different grinding parameters were discussed. Finally, in order to verify the actual enhancement effect of the laser-assisted grinding process on the edge quality and machinability of the tools, turning experiments of CFRP rods were carried out using the ground tools. By observing and analyzing the surface quality of the turned CFRP bar, the advantages of laser-assisted grinding in enhancing edge quality and improving the machining quality of CFRP bars were further confirmed.

## 2. Experimental Section

### 2.1. Workpiece Parameters

**Figure 1:**
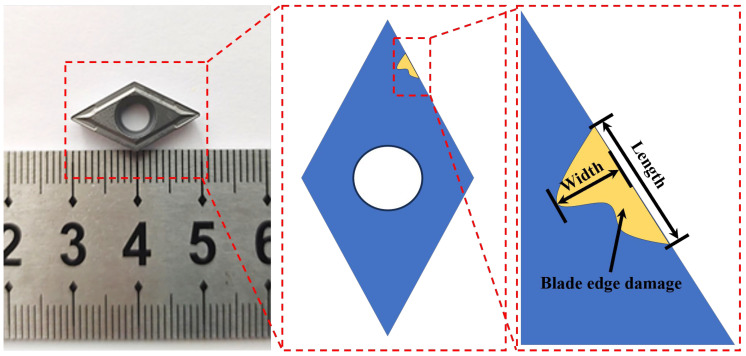
Physical drawing of the tool and schematic diagram of blade edge damage measurement.

The cutting tool material employed in this paper is WC–Co cemented carbide (Zhuzhou Cemented Carbide Cutting Tools Co., Ltd., Zhuzhou, China). WC–Co cemented carbide is frequently used as a cutting tool material owing to its exceptional hardness, high strength, outstanding wear resistance, low coefficient of thermal expansion, and increased toughness. Refer to Table 1 for the composition of the WC–Co cemented carbide blank tool used in this study. The dimensions of the WC–Co cemented carbide blank tool are 20 mm × 10 mm × 4 mm, with a 0.4 mm machining allowance on the back surface of the blade, as shown in Figure 1. The blade edge damage after the grinding process is also illustrated in this figure. The T800H cylindrical carbon fiber (Hongwangxin Fiber Products Co., Ltd., Ji’an, China) composite workpiece has a length of 50 cm and a diameter of 20 mm. The material parameters are shown in Table 2.

### 2.2. Experimental Procedures

#### 2.2.1. Texture Pattern Design

**Figure 2:**
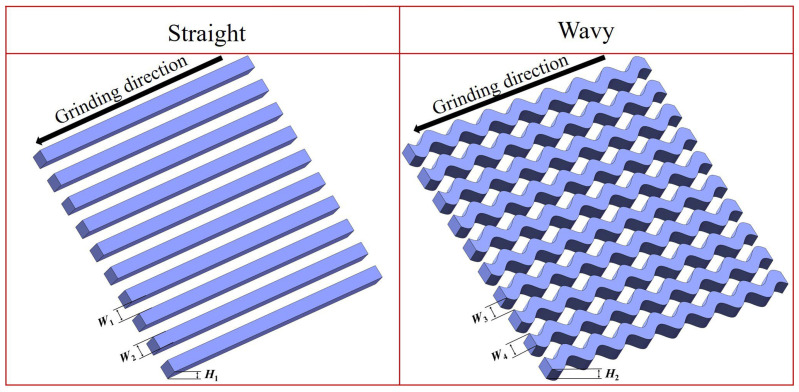
Three-dimensional structural models of the straight pattern and the wavy pattern.

In this study, two distinct laser-textured patterns were employed, as illustrated in Figure 2. The pattern on the left comprises straight texture, whereas the one on the right features wavy texture. These lines align with the wheel feed direction. The dimensions of the straight texture are shown in Table 3, and Table 4 displays the specific dimensions of the wavy-textured pattern. It has been shown that the synergistic interaction between laser-machined micro- and nano-textured structures and grinding fluids has a good heat dissipation effect. Additionally, the concave texture serves as a reservoir for the grinding fluid, offering a buffering effect. Simultaneously, it establishes a pathway for the removal of abrasive chips [25].

#### 2.2.2. Laser Texturing

**Figure 3:**
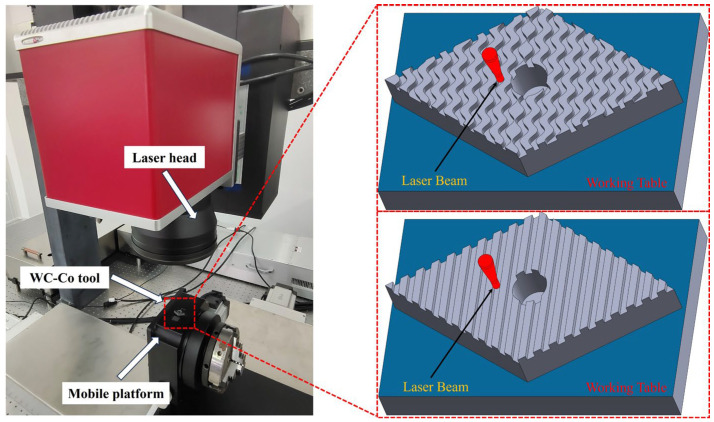
Schematic of laser texturization.

Laser processing is an advanced non-contact processing technology that utilizes the thermal effect of a high-energy-density laser beam on the surface of the workpiece to achieve precise processing of the material [26]. In this study, an ultrafast picosecond pulsed laser system model BC-2900 (Zhongshan Initialase Technologies Co., Ltd., Zhongshan, China) was used to etch the surface of a WC–Co carbide tool, including straight and wavy textures. The laser processing parameters are shown in Table 5 The experimental setup for laser processing, depicted in Figure 3, features an ultrafast picosecond pulsed laser with an energy distribution adhering to the Gaussian distribution law. The Gaussian laser beam is modulated by a system of mirrors and transformed into a flat-top laser beam. During texturing, this flat-top laser beam exhibits the advantages of uniform top energy distribution and clear edge characteristics, allowing for more precise cutting operations, resulting in sharper and more accurate edges. Following the laser texturing procedure, the tools underwent ultrasonic cleaning in anhydrous ethanol for a duration of 10 min. Subsequently, the cleansed tools were blow-dried using a nitrogen gas gun. Finally, in order to observe the microstructure, we carried out a comprehensive surface morphology observation with the help of a laser confocal microscope to analyze the cross-section height profile of the laser-textured WC–Co carbide tools.

#### 2.2.3. Grinding Experiments

**Figure 4:**
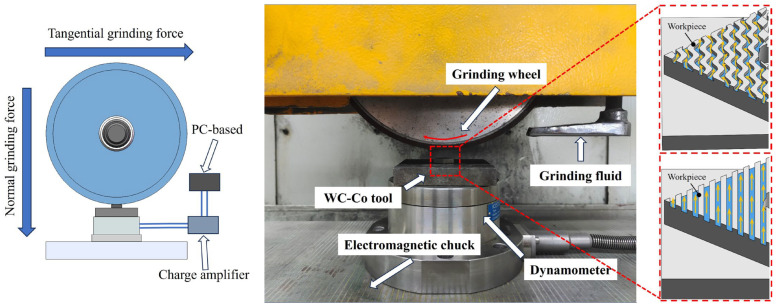
Schematic diagram of grinding experiment.

**Figure 5:**
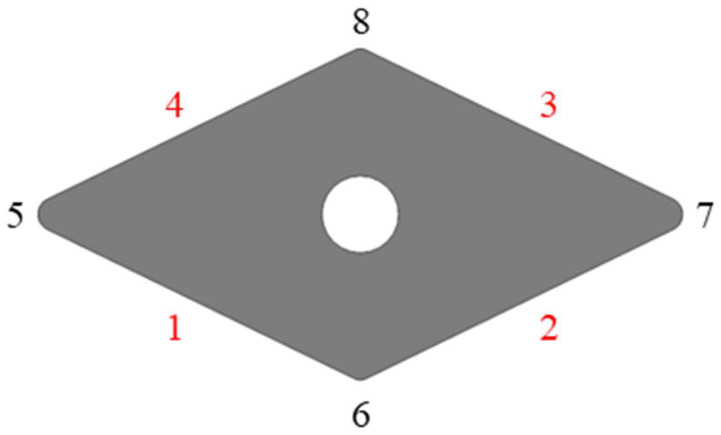
Blade grinding sequence.

The plane grinding tests on WC–Co carbide inserts were performed utilizing a CNC high-precision plane grinder (MGK7120, Hangzhou Machine Tool Factory, Hangzhou, China). The grinding schematic is shown in Figure 4. The machining surface of the insert includes four flat surfaces and four arc surfaces. If the inserts are processed in the order of alternating flat surfaces and rounded surfaces, on the one hand, the positions of the grinding wheels and inserts need to be adjusted repeatedly, and on the other hand, the material on the flat surfaces will be removed when processing the rounded surfaces, which makes it difficult to ensure the processing quality. Therefore, under the production process, the machining sequence shown in Figure 5 is used to grind the inserts. Processing entails the first processing of all planes, and then the entire arc surface, including the processing of each plane to the preset size through the fixture to form the blade rotation, to achieve the sequential processing of each plane. After machining all planes, chamfering is performed. Whenever a blade is finished, the grinding wheel is trimmed to ensure the quality of machining. Experimental conditions for plane grinding are outlined in Table 6. The results of the study showed that the coarser the grit size of the grinding wheel, the higher the grinding stress. In this study, resin-bonded diamond grinding wheels were selected and were balanced, dressed, and sharpened prior to use. During the experiments, it was ensured that the grinding wheel and tool work area received a continuous supply of water-based coolant at a concentration of 3% at a rate of 25 L per minute. In order to measure the tangential and normal grinding forces, a high-precision force gauge (Kistler 9272, KISTLER, Winterthur, Switzerland) was placed under the tool. The force measurement system for this test consisted of the following devices: force gauge, charge amplifier, and XYZ adapter cable. The force measurement system consists of a force gauge mounted next to the fixture, which receives a signal that is processed by the charge amplifier and transferred by the adapter cable to the PC, where the force signal is analyzed by the HRSO DW software (Oracle 12C). Five sets of normal and tangential grinding force data were measured throughout the grinding experiment and processed using the averaging method. A laser confocal microscope (LEXT OLS5000, OLYMPUS, Tokyo, Japan) was used to observe the surface morphology and chipping angle of the tool and to assess the surface roughness of the tool.

#### 2.2.4. Cutting Experiments

**Figure 6:**
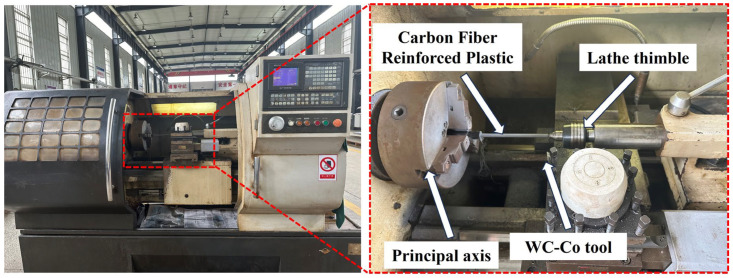
Cutting experiment diagram.

The cutting experiments were carried out on a CAK40100v1 CNC horizontal lathe (Shenyang Diyi Machine Tool Manufacturing Co., Ltd., Shenyang, China). The machining setup is shown in Figure 6. During machining, the CFRP bar is clamped in a customized fixture which is connected to the machine spindle. Turning on CFRP rods was carried out using ground WC–Co carbide tools, and the experimental conditions for turning are shown in Table 7. The cutting was carried out under dry conditions without using any cutting fluid. The turned CFRP rods were observed using a super-depth-of-field microscope (VHX-5000, KEYENCE, Osaka, Japan) to compare the effect of carbide tools with different edge defects on the surface of CFRP rods. The tool wear after turning was also observed.

## 3. Results and Discussion

### 3.1. Surface Morphology of Laser Texturing

**Figure 7:**
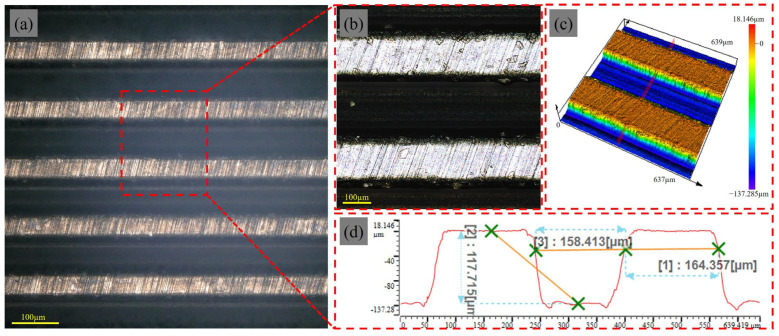
Surface morphology of straight texture after laser texturing: (**a**) overall surface morphology; (**b**) local surface morphology; (**c**) 3D surface morphology; (**d**) cross-section profile curve.

Figure 7a illustrates the overall surface morphology of the straight texture, where the tool surface appears to have a regular straight texture due to laser ablation. Figure 7b,c depicts localized surface features and 3D height maps of this straight texture. The flat-topped picosecond laser beam produced sharp laser boundaries that clearly delineated the treated and untreated areas. It is worth noting that the ultrashort pulse duration of the picosecond laser allows for rapid vaporization of the tool material, thus minimizing thermal damage to the tool [27]. Figure 7d illustrates a cross-sectional profile of the straight texture. The width of the grooves of a single straight texture unit is 158.413 µm, the spacing is 164.357 µm, and the depth is 117.715 µm. Due to the scanning path of the laser, the beam spends more time in the front and rear areas of the bottom. These areas are exposed to the laser for a longer period of time, resulting in more heat build-up, which produces greater thermal expansion and dimensional changes. This thermal effect results in a slight deviation from the theoretical dimensions, but the deviation is still within 20 microns.

**Figure 8:**
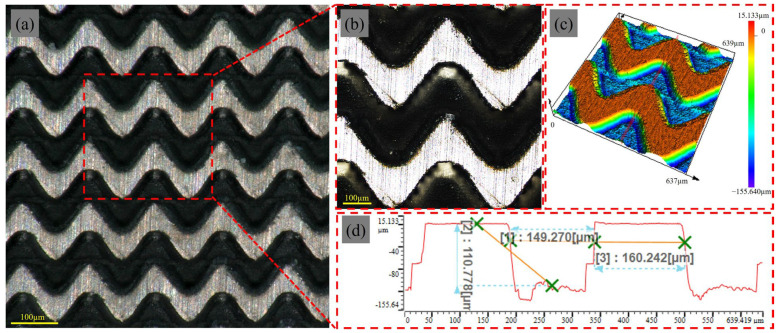
Surface morphology of the wavy texture after laser texturing: (**a**) overall surface morphology; (**b**) local surface morphology; (**c**) three-dimensional surface morphology; (**d**) cross-section profile.

Figure 8a shows a regular wave weave due to material removal, with a small amount of recast layer produced on the surface and bottom relative to the straight weave. Figure 8b shows a small amount of recast layer produced on the unmachined surface due to the laser beam staying in the corner for too long. In Figure 8c, it can be clearly observed that the grooves produced by the laser processing of the WC–Co carbide tool workpiece are not absolutely smooth, and the majority of the wave-shaped grooves produced by the tool are areas of recast layer, due to the complexity of the structure of the wave weave itself, and the laser beam will be shifted during the process of ablation of the surface, which results in the distribution of the laser energy and the absorption of the laser energy becoming uneven. This results in certain areas receiving too much energy, which triggers the recasting phenomenon. Figure 8d shows a single wave weave with slot width and spacing of 149.270 μm and 160.242 μm, respectively, and a depth of 110.778 μm.

### 3.2. Comparative Analysis of Grinding Forces

**Figure 9:**
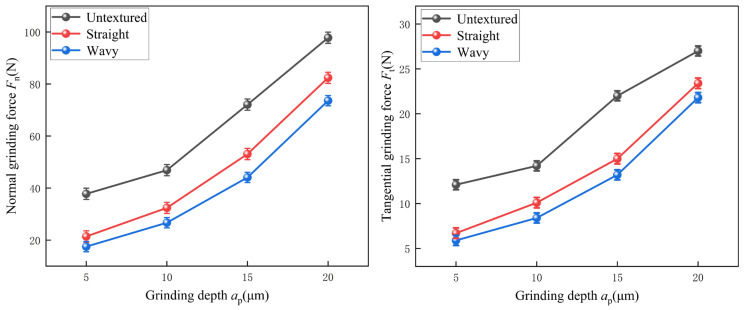
Effect of different grinding depths on normal and tangential grinding forces.

Grinding force is a crucial consideration in the grinding process. It directly affects the degree of tool deformation during machining, which in turn relates to machining accuracy and tool quality [28]. Figure 9 clearly shows the comparison of tangential and normal grinding forces for the untextured, straight, and wavy-textured configurations at different depths of grinding. Comparing the grinding forces of the straight- and wavy-textured configurations at different grinding depths, we observe that there are slight differences between the two. Specifically, the maximum difference in normal grinding force between straight and wavy texture is 9 N, and the minimum difference is 3.9 N. The maximum difference in tangential grinding force is 1.8 N, and the minimum difference is 0.8 N. Comparing these two types of grinding forces, the wavy texture shows a superior performance to the straight texture. This is mainly due to the tiny raised and depressed structures formed by the wavy texture on the tool surface. These structures act as heat dispersal points and stress buffers during the grinding process, effectively reducing the concentration of heat and stress generated by grinding and thus reducing the grinding force. Further comparing the structured tools (including straight- and wavy-textured) with the untextured tools, we find that there is a significant difference in the grinding forces between the two. Compared to the untextured tools, the straight texture reduced the normal grinding force by 15.7% to 43.4% and the tangential grinding force by 13.3% to 44.6%. The wavy texture, on the other hand, performed even better in terms of grinding force reduction, with reductions in normal grinding forces ranging from 24.7% to 53.7% and tangential grinding forces ranging from 19.3% to 51.2% compared to the untextured tools. There are three main reasons for these significant differences. Firstly, the structured design of the tool surface reduces the actual contact area between the tool and the grinding wheel, which in turn reduces the tangential and normal grinding forces during the grinding process. Secondly, these microstructures can effectively spread the heat and stress generated during the grinding process over a larger area, avoiding excessive concentration of heat and stress at a single point and further reducing the grinding force. Finally, the textured tool is able to store cutting fluid [29], which enhances the role of the cutting fluid in the grinding process and helps to reduce the coefficient of friction between the tool and the grinding wheel, thus further reducing the grinding force. As the grinding depth deepens, the normal grinding force rises accordingly at the same feed rate and wheel speed. At the same time, the chip thickness of individual grits increases, leading to an increase in their material removal capacity and force, and therefore an increase in the overall grinding force.

**Figure 10:**
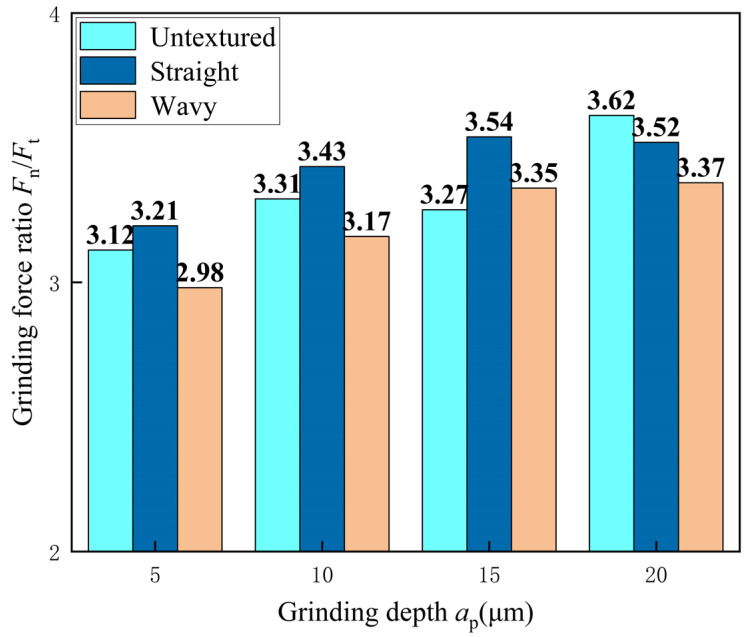
Effect of different grinding depths on grinding force ratio.

Figure 10 reveals the differences in the grinding force ratios between the untextured, straight, and wavy-textured tools at different depths of grinding. The grinding force ratios between the three profiles show an overall increasing trend. The grinding force ratios of the untextured tools ranged from 3.12 to 3.62, whereas the grinding force ratios of the wavy-textured tools were in the range of 2.98 to 3.37. This difference may be due to the wavy texture enhancing the flow of coolant within the grinding zone, so that the coolant penetrates the grinding zone more efficiently, which reduces the grinding temperature as well as the friction and heat build-up during the process, which ultimately leads to a decrease in the grinding force ratio. However, the grinding force ratio is higher for straight-textured tools than for untextured tools. Figure 7 shows that the grinding forces in the tangential and normal directions are lower for the straight-textured tool than for the untextured tool. The reason for this can be deduced from the fact that under the influence of the straight texture, the normal force is reduced to a lesser extent as compared to the tangential force. This is because the straight textures are aligned primarily along the direction of grinding (tangential), so they primarily change the contact area in the tangential direction. In contrast, the contact area perpendicular to the grinding direction (normal) is less affected by the straight texture.

### 3.3. Surface Morphology After Grinding

**Figure 11:**
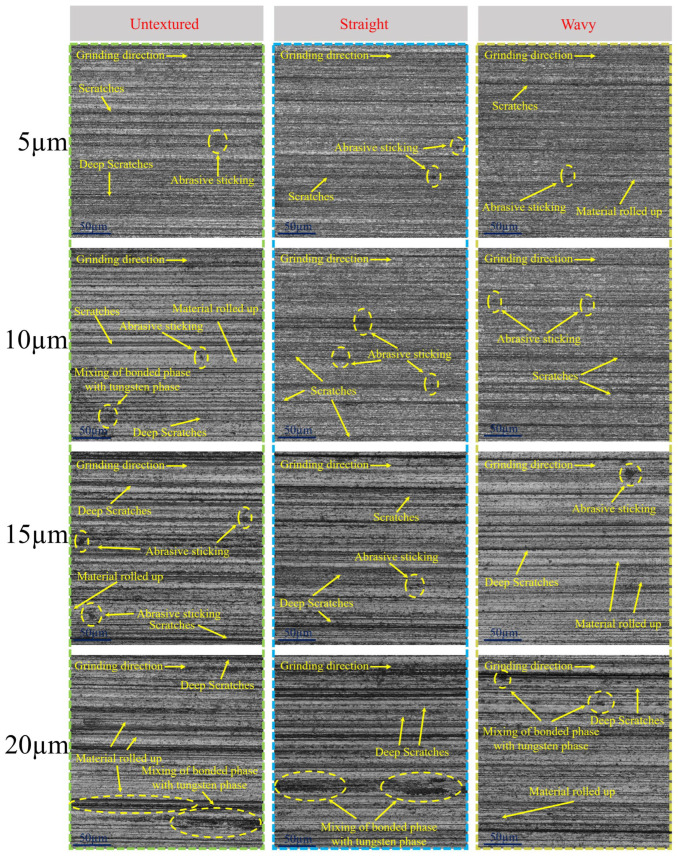
Surface topography at different grinding depths.

Due to the high hardness and brittleness of WC–Co cemented carbide, the material removal process mainly presents a brittle fracture mode [30]. Figure 11 shows the surface morphology of a WC–Co carbide tool, which exhibits two distinct wear characteristics: one wear trace is larger in size and has a significant depth, which is referred to as a “coarse wear trace”, while the other is smaller in size and has a shallower depth, which is defined as a “fine wear trace”. Comparison of the surface condition of the untextured tool with that of the wavy tool at a grinding depth of 5 µm shows that the untextured tool clearly shows coarse wear marks on the surface. Due to the failure of the abrasive chips generated during the grinding process to be discharged in a timely manner, a large area of abrasive chip adhesion appeared on the surface of the untextured tool. In contrast, the number of fine wear marks on the surface of the textured tool was small, and the area of abrasive chip adhesion was also relatively small. When the grinding depth was increased to 10 µm, the untextured tool was subjected to flow and roll-up of material under the action of abrasive particles due to the high grinding force during the grinding process. At the same time, the bonded-phase Co was partially melted at high temperatures, and the hard-phase WC was broken due to thermal stresses, as the grinding heat was not transferred away through the grinding fluid in time. These changes make the hard and bonded phases mix more closely on the grinding surface, forming an intermingled appearance. In the case of the textured tool, the overall condition of the surface was still better than that of the untextured tool, although more fine abrasive marks appeared on the surface when the grinding depth was increased. Further increasing the grinding depth to 15 µm, the surface of the untextured tool showed more coarse abrasions, while a large amount of crystal powder adhered due to crystal breakage. The surface of the textured tool also started to show more coarse wear marks, but the brittle removal from the surface of the straight-textured tool resulted in only a small amount of fragmentation. Coarse wear marks and material adhesion on the surfaces of the wavy-textured tools were relatively few. At grinding depths up to 20 µm, coarse wear marks and mixing of the tungsten phase with the bonded phase were observed on the surfaces of both the untextured and textured tools. However, the area of the tungsten-phase and bonded-phase mixing region on the surface of the wavy-textured tool was smaller compared to the other two tools. This indicates that during the grinding process, the microchannel structure of the wavy-textured tool effectively promotes the flow of the grinding fluid, which carries away a large amount of grinding heat and reduces the temperature of the tool surface.

**Figure 12:**
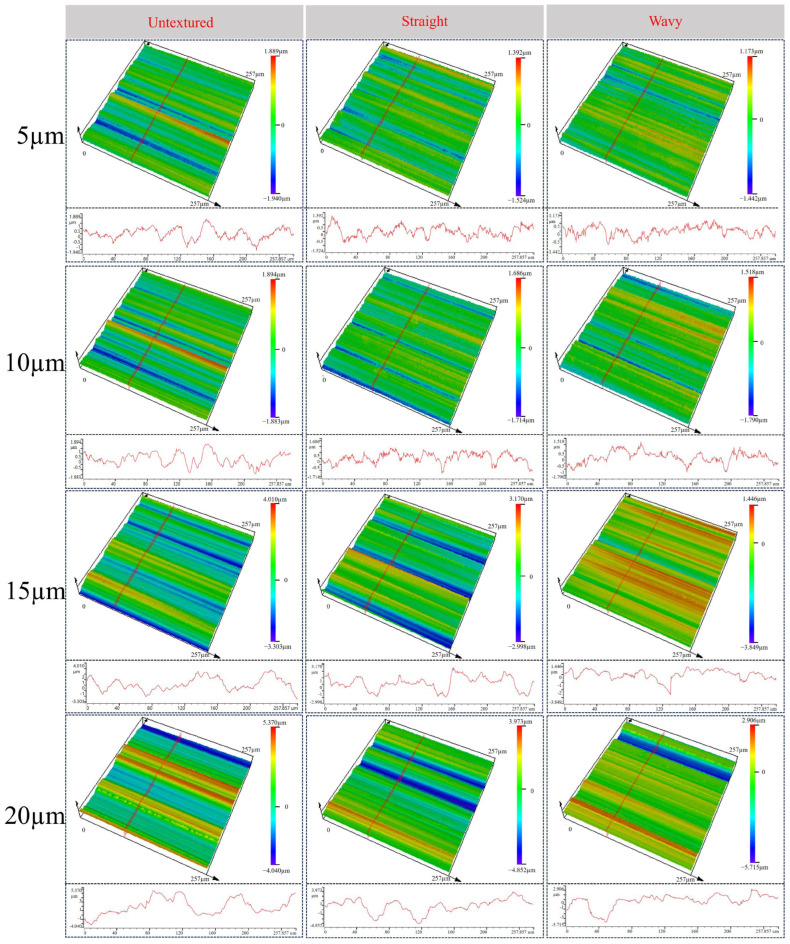
Surface height profiles at different grinding depths.

Figure 12 shows the three-dimensional morphology of the surface of the WC–Co carbide tool after grinding at grinding depths of 5, 10, 15, and 20 µm. From the color distribution of the 3D surface, it can be clearly found that scratches of different widths and depths are randomly distributed on the surface due to the action of abrasive grains. With the increase of the grinding depth, the color of the blue valley gradually becomes deeper and wider, indicating that the depth and width of the scratches are also increasing. At larger grinding depths, the WC–Co carbide tools are more prone to brittle fracture [31], and the deterioration of surface quality increases. Comparison of the 3D morphology maps of tools with and without texture shows that the surface of tools with texture has shallower scratches and less abrasive chip adhesion. This is due to the presence of surface texture; the actual area of grinding wheel grinding is reduced, which in turn reduces the grinding force. In addition, the presence of microfluidic channels on the surface makes it easier for the abrasive chips to be discharged and avoid being reworked by the grinding wheel.

**Figure 13:**
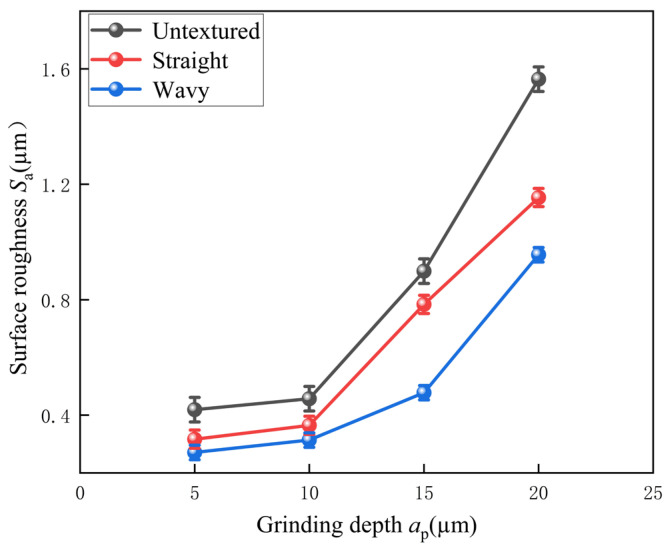
Surface roughness at different grinding depths.

Regarding the surface roughness, the data obtained using laser confocal microscopy are shown in Figure 13. Surface roughness is an important indicator of surface quality, with smaller values indicating a smoother surface [32]. It was observed in the experiments that the surface roughness of all three tools showed an increasing trend as the depth of grinding increased. This phenomenon is attributed to the fact that the contact area between the abrasive grain and the workpiece surface increases with the depth of grinding, which results in deeper scratches and greater deformation on the workpiece surface, ultimately leading to an increase in surface roughness. In the range of grinding depth from 5 µm to 20 µm, the surface roughness increased by 73.2% for untextured tools, 72.5% for straight-textured tools, and 71.7% for wavy-textured tools. In addition, the experiments also compared the differences in surface roughness between textured and untextured tools under different grinding depth conditions. The results showed that the straight-textured tool achieved 24.3%, 20.1%, 12.8%, and 26.2% reduction in surface roughness compared to the untextured tool for the four set depths of grinding, respectively, while that of the wavy textured tools decreased by 35.3%, 31.3%, 46.8%, and 38.9%, respectively. These results indicate that after grinding, the tools with micro-textured structure have better surface quality compared to the untextured tools. This advantage is mainly due to the geometrical change of the tool surface by the texture, which can effectively reduce the grinding force during the grinding process and reduce the local stress concentration phenomenon. At the same time, the texture creates small flow channels on the tool surface, which makes it easier for the grinding fluid to penetrate and distribute, so that grinding debris can be flushed away in time to avoid adhering to the tool surface. The surface roughness after grinding of the straight- and wavy-textured tools was further compared. The better surface quality of the wavy-textured tool is mainly attributed to the undulating pattern of the wavy texture, which is more favorable to the penetration and uniform distribution of the grinding fluid, thus effectively reducing the grinding temperature, reducing thermal damage, and more effectively flushing away the grinding chips. In addition, the undulation of the wavy texture also makes the contact area between the grinding wheel and the tool surface and the stress distribution more uniform, which helps to reduce the grinding scratches or micro-cracks caused by stress concentration, and thus reduce the surface roughness. In contrast, a straight-textured profile can lead to stress concentrations in certain areas, which can increase surface roughness.

### 3.4. The Edge Damage Condition of Cutting Blades After Grinding

**Figure 14:**
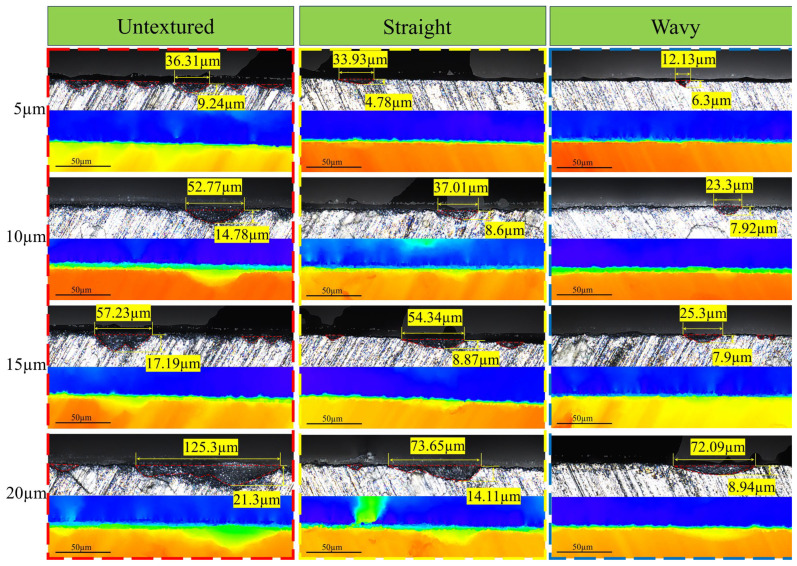
The edge damage condition of cutting blades after grinding.

Figure 14 shows the chipping of the cutting edges of three types of tools after grinding with resin-bonded diamond wheels at different grinding depths. As the grinding depth increases, the length and depth of edge chipping increase. According to the standards, the length of edge chipping should be less than or equal to 50 µm, and the depth should be less than or equal to 45 µm. The optimum grinding results were observed on the wavy-textured tool with a grinding depth of 5 µm, where the length of edge chipping was 12.13 µm and the depth of edge chipping was 6.3 µm. However, on the untextured tool with a grinding depth of 20 µm, the length of edge chipping reached 125.3 µm and the depth 21.3 µm, which is far beyond the standard requirements. According to Li et al. [33], the excessive grinding volume and grinding force of WC–Co cemented carbide tools during wheel machining can lead to the extrusion and detachment of a large number of WC crystals, which in turn leads to the formation of large chipping at the tool edges. Therefore, excessive grinding volume and grinding force are considered to be the main causes of edge chipping. The edges of textured tool edges are mostly presented as single tiny cracks, while the edges of untextured tool edges are mostly jagged continuous cracks and obtuse cracks. The non-conforming edge of the untextured tool was found to be substandard at a grinding depth of 10 µm, whereas the non-conforming edge of the weave tool was found to be substandard only at a grinding depth of 20 µm. According to the above discussion, the presence of texture can effectively reduce the grinding force generated during grinding, which in turn reduces the large amount of WC crystals being extruded and dislodged, and thus ensures the quality of the edge. The microfluidic channel on the tool surface can discharge the grinding chips in time, which can prevent the grinding chips from accumulating and scratching the tool surface during the grinding process to a certain extent, thus reducing the risk of edge chipping.

### 3.5. Surface Morphology of CFRP Bars After Cutting

**Figure 15:**
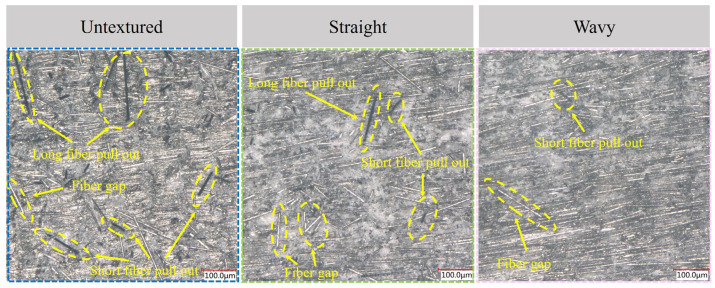
Surface morphology of CFRP bars after turning with different tools.

Figure 15 shows a comparison of the surface morphology of CFRP bars after turning them with tools having different edge chipping conditions at a set depth of 20 microns for a single grinding pass. Overall, the surface morphology of CFRP bars processed with laser-assisted grinding tools shows significant improvement after grinding. In the turning experiments, the large edge chipping defects resulted in poor surface quality of CFRP rods with exposed carbon fibers and a large number of long craters, which were widely distributed [34]. For the laser-assisted ground tools, the edge chipping was relatively diminished and the surface quality of the turned CFRP rods was better, with less fiber pull-out and fewer fiber voids. This is because CFRP is a composite material made of carbon fiber and resin matrix, and the bonding force between CFRP fiber and matrix is relatively weak. During the cutting process, if there is chipping on the tool edge, it is easy to separate the fiber from the matrix, resulting in fiber pull-out and void generation. Therefore, laser-assisted grinding technology can improve the machined surface quality of CFRP by optimizing the quality and stability of the tool edge.

## 4. Conclusions

In this paper, an experimental study of laser-assisted grinding for machining of WC–Co carbide tools has been carried out. Laser-textured tools were demonstrated and their performance was analyzed in comparison with untextured tools. The study provides an insight into the effectiveness of laser-assisted grinding in optimizing the surface quality of WC–Co tungsten carbide tools, mitigating edge chipping and reducing grinding forces. Synthesizing the various aspects of the above experiments and studies, the following conclusions were drawn. By controlling the laser processing parameters, straight textures and wavy textures were formed on the surface of the WC–Co carbide tool. Moreover, there was no fracture or cracking on the groove edges and less of a recast layer at the bottom of the groove.

By comparing conventional grinding with laser-assisted grinding on WC–Co carbide tools, we found that, under the same working conditions, the maximum reduction in normal grinding force of the wavy-textured tool reached 53% and the maximum reduction in tangential grinding force was 51.2%. The reduction in grinding force is mainly attributed to the reduction in material hardness after laser ablation and the design of the wavy texture, which helps in chip evacuation, thus reducing wear and friction during the grinding process.

As a two-phase composite material, tungsten–cobalt alloy was observed to undergo an increase in the depth and number of scratches with the gradual increase in the depth of grinding, while the mixing area between the bonded and tungsten phases was gradually enlarged, with a corresponding increase in the amount of abrasive chip attachment. However, the surface morphology of the textured tool exhibited a superior state compared to the untextured tool. Specifically, the straight-textured tool reduced the surface roughness by up to 26.2% compared to the untextured tool, while the wavy-textured tool performed better, reducing the surface roughness by up to 38.9% compared to the untextured tool.

The damage to the blade after grinding primarily appears as individual minute cracks, serrated continuous cracks, and blunt cracks. Especially when the grinding wheel processes materials with concave edges, the cracks extend from the WC phase to other WC grains, resulting in a large number of WC grains being extruded. These extruded WC grains are eventually dislodged, resulting in the formation of large notches. When comparing untextured and textured tools, by the time the grinding depth reaches 10 µm, the edge of the untextured tool is already out of specification; for the textured tool, this does not occur until the grinding depth reaches 20 µm. This suggests that laser texturing technology can dramatically improve tool performance in deep machining and adapt to more complex manufacturing needs.

The surface morphology characteristics of CFRP rods were analyzed by comparing the turning tests performed on tools after laser-assisted grinding and conventional grinding. After laser-assisted grinding, the tool edge chipping was smaller and the surface quality of the turned CFRP rods was better, with less fiber pull-out and fewer fiber voids. After conventional grinding, the tool edge chipping was larger, and the exposed carbon fibers were larger in size and widely distributed, resulting in poorer surface quality of CFRP rods.

## Figures and Tables

**Table 1:** Chemical composition and properties of WC–Co material.

WC (wt%)	Co (wt%)	Hardness (HV)	Density (g/cm^3^)	Grain Size (mm)
94	6	1700	14.9	0.7

**Table 2:** Physical properties of CFRP.

Parameter	Value
Length *l* (cm)	50
Diamater *Φ* (mm)	20
Density *ρ* (g/cm^3^)	1.81
Diameter *d*_f_ (mm)	20
Longitudinal modulus *E*_1_ (GPa)	294
Transverse modulus *E*_2_ (GPa)	19.6
Shear modulus *G*_12_ (GPa)	103

**Table 3:** Parameters of straight pattern.

Pattern	*W*_1_ (µm)	*W*_2_ (µm)	*H*_1_ (µm)
Straight	150	150	120

**Table 4:** Parameters of wavy pattern.

Pattern	*W*_3_ (µm)	*W*_4_ (µm)	*H*_2_ (µm)
Wavy	150	150	120

**Table 5:** Parameters for laser machining of WC–Co carbide tools.

Parameter	Value
Focal length *f* (mm)	135
Pulse frequency *f*_p_ (kHz)	60
Average laser power *P*_avg_ (W)	9.5
Scan speed *v*_ss_ (mm/s)	10
Scanning spacing *d*_ss_ (μm)	5
Number of laser ablation *N*	8

**Table 6:** Parameters used for grinding WC–Co carbide tools.

Parameter	Value
Tools	Resin-bonded diamond grinding wheel
Type of grinding wheel	200 × 20 × 32 SD100
Grinding mode	Down-grinding
Grinding coolant	Water-based coolant (Type W20)
Grinding speed *V*_s_	30 m/s
Workpiece speed *V*_w_	2000 mm/min
Depth of grinding *a*_p_	5, 10, 15, 20 μm

**Table 7:** Parameters used for cutting CFRP bar.

Parameter	Value
Cutting feed *f* (mm/r)	0.1
Cutting speed *n* (r/min)	60
Cutting depth *a*_p_ (mm)	0.3

## Data Availability

The original contributions presented in the study are included in the article; further inquiries can be directed to the corresponding author.
